# Preferred Treatment and Expected Risk in Coronary Intervention Patients With Peripheral Arterial Disease: Cardiologists’ Views Versus Trials Data

**DOI:** 10.1016/j.cjco.2025.03.018

**Published:** 2025-03-31

**Authors:** Tineke H. Pinxterhuis, Clemens von Birgelen, Eline H. Ploumen, Daphne van Vliet, Marlies M. Kok, Rosaly A. Buiten, Liefke C. van der Heijden, Paolo Zocca, Carine J.M. Doggen

**Affiliations:** a Department of Cardiology, Thoraxcentrum Twente, Medisch Spectrum Twente, Enschede, The Netherlands; b Health Technology and Services Research, Faculty BMS, Technical Medical Centre, University of Twente, Enschede, The Netherlands; c Clinical Research Centre, Rijnstate Hospital, Arnhem, The Netherlands

## Abstract

**Background:**

In aging Western populations, there is an increase in the prevalence of both coronary artery disease and peripheral arterial disease (PADs). Treatment of patients who undergo percutaneous coronary intervention (PCI) and have concomitant PADs may pose a challenge, but preferences of cardiologists regarding treatment and their views on complication risks are unknown.

**Methods:**

This work was a survey-based study comparing cardiologists’ views with patient-level data of PCI trials (BIO-RESORT and BIONYX).

**Results:**

The survey was completed by 47 of 208 (23%) invited cardiologists. A growing prevalence of PADs was observed in the trials and by 50% of the respondents. Cardiologists estimated that 22% of current PCI patients had PADs, whereas this rate was 7.3% among 6002 all-comer patients. In PADs patients, PCI procedural complication rates were estimated to be higher, which was not observed in either trial. The estimated higher 3-year rates of bleeding, myocardial infarction, and cardiac death in PCI patients with PADs were corroborated by corresponding trial data (*P* = 0.014, *P* = 0.005, and *P* < 0.001, respectively). Nevertheless, PADs affected the preferred treatment in many cardiologists, and cardiologists held disparate views on preferred coronary treatment.

**Conclusions:**

Cardiologists correctly estimated the increase in PADs over time among PCI patients, but they overestimated the current prevalence. An increased risk of adverse clinical events after PCI was correctly recognized, and concomitant PADs often affected the preferred treatment. Notably, cardiologists held disparate views on optimal preferred coronary treatment, which may be attributed to the lack of reported data about this patient group. Thus, more clinical attention and research is warranted.

## Graphical abstract

**Figure:**
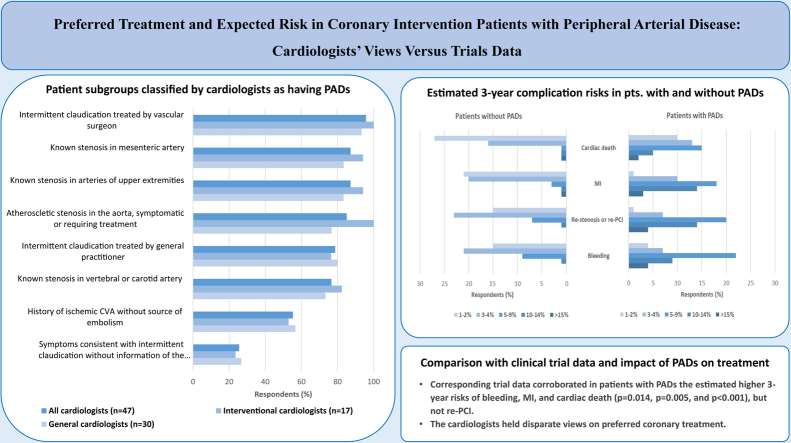

## Résumé

**Contexte:**

Dans les populations occidentales vieillissantes, on observe une augmentation de la prévalence des maladies coronariennes et des maladies artérielles périphériques (MAP). Le traitement des patients qui subissent une intervention coronarienne percutanée (ICP) et qui présentent une MAP concomitante peut constituer un défi, mais les préférences des cardiologues en matière de traitement et leur point de vue sur les risques de complications sont inconnus.

**Méthodologie:**

Une étude basée sur un sondage comparant les opinions des cardiologues avec les données des essais cliniques d'ICP au niveau des patients (BIO-RESORT et BIONYX).

**Résultats:**

Le sondage a été complété par 47/208 (23 %) des cardiologues invités. Une prévalence croissante des MAP a été observée dans les essais et par 50 % des répondants. Les cardiologues ont estimé que 22 % des patients actuellement soumis à une ICP souffraient d'une MAP, alors que ce taux était de 7,3 % parmi les 6 002 patients de tous horizons. Chez les patients atteints de MAP, les taux de complications procédurales de l'ICP étaient estimés plus élevés, ce qui n'a pas été observé dans les deux essais. Des taux estimés plus élevés d'hémorragie, d'infarctus du myocarde et de décès cardiaque à 3 ans chez les patients atteints de MAP ont été corroborés par les données des essais correspondants (*p*=0,014, *p*=0,005 et *p*<0,001, respectivement). Néanmoins, les MAP ont influencé le traitement préféré par de nombreux cardiologues, et les cardiologues avaient des opinions divergentes sur le traitement coronarien à privilégier.

**Conclusions:**

Les cardiologues ont correctement estimé l'augmentation des MAP au fil du temps chez les patients ayant subi une ICP, mais ils ont surestimé la prévalence actuelle. Le risque accru d'événements cliniques indésirables après l'ICP a été correctement reconnu, et les MAP concomitantes ont souvent affecté le traitement privilégié. En particulier, les cardiologues avaient des opinions divergentes sur le traitement coronarien optimal préféré, ce qui peut être attribué à un manque de données sur ce groupe de patients qui mériterait plus d'attention clinique et justifie des recherches futures.

Coronary artery disease (CAD) and peripheral arterial disease (PADs) are both manifestations of atherosclerosis and have similar cardiovascular risk factors.^1^ As life expectancy increases, the incidence of both CAD and PADs is expected to rise. In the United States, the number of patients with CAD is expected to rise from 11.7 million to 17.3 million by 2040,^2^ with a similar increase (by 40%-50%) expected in The Netherlands.^3^^,^^4^ In addition, the prevalence of PADs has increased and is expected to further grow.^4^^,^^5^ Consequently, the prevalence of patients with obstructive CAD who also suffer from PADs will continue to increase.

Research has shown that patients who undergo percutaneous coronary intervention (PCI) and have concomitant PADs have inferior outcomes, with increased risks of mortality, repeated coronary revascularization, and bleeding.^6^, ^7^, ^8^, ^9^

In addition, PADs is often underdiagnosed and undertreated, also the case in patients with CAD.^10^^,^^11^ PCI patients with concomitant PADs should be considered a high-risk subgroup that benefits from tailored medical treatment and more strict secondary prevention. Yet, data are lacking regarding cardiologists' views on treatment preferences and complication risks in patients with obstructive CAD and concomitant PADs.

Therefore, in this study we aimed to assess the views of cardiologists on: 1) the estimated prevalence of concomitant PADs among PCI patients; 2) their preferred treatment strategy in these patients; and 3) the expected complication risk in PCI patients with or without PADs. The findings from this questionnaire-based assessment were then compared with a pooled analysis of individual patient–level data from 2 large-scale PCI trials involving all-comers that recorded data on the presence or absence of known PADs.

## Methods

### Study design

In November 2022, cardiologists at 22 hospitals in The Netherlands were invited to answer an online questionnaire (English version, see Supplemental Appendix S1) specifically developed for this study. To optimize the phrasing and order of the questions, the questionnaire was first tested among nonparticipating cardiologists at the organizing centre (Medisch Spectrum Twente, Enschede, The Netherlands). Based on this experience, the questionnaire was refined before cardiologists were invited to respond.

The first part of the questionnaire assessed the characteristics of the respondents. The second part evaluated which patients were classified as “having PADs” as well as the estimated prevalence of PADs in PCI patients. The third part assessed the respondents’ views on the impact of PADs on treatment preferences and complication risks during and after PCI. One clinical case was outlined to investigate the cardiologists’ views on the impact of PADs on their choice of coronary revascularization.

### Data analysis

The results of the questionnaire were compared with the results of a pooled patient-level data analysis from 2 large-scale, randomized, drug-eluting stent trials (Comparison of Biodegradable Polymer and Durable Polymer Drug-Eluting Stents in an All-Comers Population [BIO-RESORT, TWENTE III, NCT01674803] and Bioresorbable Polymer-Coated Osiro Versus Durable Polymer-Coated Resolute Onyx Stents [BIONYX, TWENTE IV, NCT02508714]). Details of the original trials have been reported previously.^12^, ^13^, ^14^, ^15^ In brief, these investigator-initiated, patient-blinded, randomized stent trials included 6002 all-comer patients who required PCI. Patients were eligible for enrollment in the trial if they were ≥ 18 years of age, able to provide informed consent, and required PCI with drug-eluting stent implantation. All coronary syndromes, *de novo* and restenotic lesions, and coronary artery or bypass lesions were permitted. There was no limit for lesion length, reference size, number of lesions, or diseased vessels to be treated. Patients from both trials were classified in the same way as having PADs if they---by anamnesis or medical records---had a history of at least 1 of the following: symptomatic atherosclerotic lesion in the lower or upper extremities; atherosclerotic lesion in the aorta causing symptoms or requiring treatment; atherosclerotic lesion in the carotid or vertebral artery related to a nonembolic ischemic cerebrovascular event; or symptomatic atherosclerotic lesion in a mesenteric artery. The BIO-RESORT trial included patients in the period from December 2012 to May 2015, whereas the consecutive trial, the BIONYX, enrolled patients from October 2015 to December 2016. Annual clinical follow-up was obtained by telephone, questionnaire, or visit to the outpatient clinic. An independent clinical research organization performed data monitoring, processing of clinical outcome data, and independent clinical event adjudication. Several types of adverse cardiovascular events were reported, including mortality (including cardiac mortality), repeated coronary revascularization, myocardial infarct, and bleeding. The trials complied with the Declaration of Helsinki. The medical ethics committee at Twente and the institutional review boards of all participating centres approved all original trials. All trial participants provided written informed consent.

### Statistical analyses

Continuous variables are presented as mean ± standard deviation. For dichotomous and categorical variables, data are expressed as frequency and percent. Categorical variables were compared using the χ^2^ test. For several comparisons, participants were classified as either interventional or general cardiologists. Results were considered statistically significant at *P* < 0.05. Statistical analysis was performed using SPSS version 28.0 (IBM Corp, Armonk, NY).

## Results

### Study participants and clinical trials

The questionnaire was sent to 22 hospitals, approaching about 208 cardiologists. A total of 22.6% (47 of 208) of cardiologists from 86.4% (19 of 22) of all hospitals responded; 74.5% (35 of 47) were male and 36.2% (17 of 47) were interventional cardiologists, performing an average of 274 ± 79 PCI procedures annually. Further characteristics are presented in Table 1. A total of 6002 all-comer PCI patients were enrolled in the BIO-RESORT and BIONYX trials, of whom 440 (7.3%) had concomitant PADs. The mean age of the trial patients was 64.0 ± 10.9 years, with 74.0% of them being male.

**Table 1:** Characteristics of responding cardiologists

Respondents	47 (100)
Male respondents	35 (74.5%)
Age, years	
< 30	0
30-39	13 (27.7%)
40-49	18 (38.3%)
50-59	9 (19.1%)
≥ 60	7 (14.9%)
Type of hospital	
Nonacademic hospital without PCI and heart surgery	18 (38.3%)
Nonacademic hospital with PCI, but without heart surgery	7 (14.9%)
Nonacademic hospital with PCI and heart surgery	18 (38.3%)
Academic hospital	3 (6.4%)
Other	1 (2.1%)
Interventional cardiologist	17 (36.2%)
Performing PCI	17 (100%)
Number of PCI performed per year, mean ± SD	274 ± 79
Years of performing PCI	
< 5	6 (35.3%)
5-9	3 (17.6%)
10-19	6 (35.3%)
≥ 20	2 (11.8%)

Data expressed as number (%) unless indicated otherwise.

PCI, percutaneous coronary intervention; SD, standard deviation.

### Estimated prevalence of PADs among PCI patients

About every second respondent (53%) noticed an increase in PCI patients with concomitant PADs during the last 5 years. The majority of respondents (63.8%, 30 of 47) estimated that < 25% of the PCI patients had PADs, whereas 23.4% (11 of 47) estimated the rate to be 25%-50%, 4.3% (2 of 47) estimated it to be > 50%, and 8.5% (4 of 47) provided no estimation. The respondents estimated the current average prevalence of concomitant PADs among PCI patients to be about 22%, which is higher than the prevalence observed in our clinical trials (7.3%). Yet, the noted increase in PCI patients with PADs was confirmed by the trial data, and a comparison of the BIO-RESORT trial with the subsequent BIONYX trial revealed an increase in the prevalence of PADs from 6.8% to 8.1%.

### Definition of PADs

**Figure 1:**
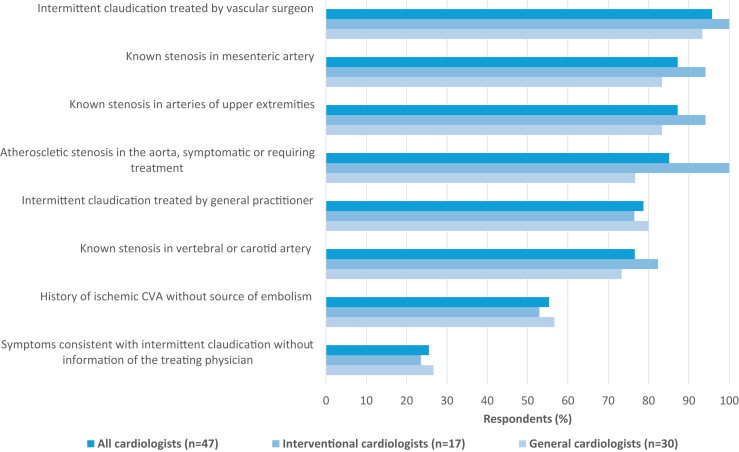
Patients classified as having peripheral arterial disease. The graph shows the percentage of cardiologists who consider the described patient groups as patients with peripheral arterial disease. CVA, cerebrovascular accident.

Cardiologists were asked which patients should be classified as having PADs. Patients with intermittent claudication treated by a vascular surgeon were classified as having PADs by 95.7% (45 of 47) of the responding cardiologists; patients with intermittent claudication treated by a general practitioner by 78.7% (37 of 47); patients with a known stenosis in the mesenteric artery as well as patients with a known stenosis in the arteries of the upper extremities by 87.2% (41 of 47); and patients with a known stenosis in the vertebral artery or carotid artery by 76.6% (36 of 47; Supplemental Table S1). Patients with symptoms suggesting intermittent claudication and patients with a history of an ischemic cerebrovascular accident without cardiac source of embolism were less frequently classified as having PADs (Fig. 1). Yet, the respondents combined different patient groups (mentioned previously) for their “individual definition” for PADs. Patients with atherosclerotic stenosis of the aorta were more often classified as PADs by the interventional cardiologists (as compared with general cardiologists), whereas there was remarkable consistency between the 2 groups of cardiologists in classifying other patient subsets as having PADs.

### Complication risk

**Figure 2:**
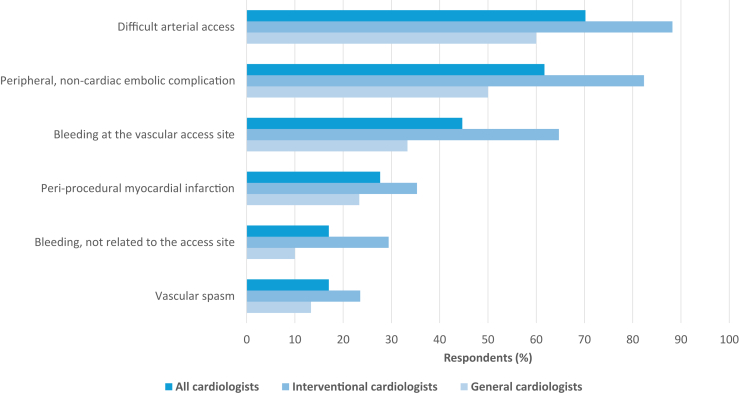
Estimated periprocedural complication risk. The graph shows the percentage of cardiologists who consider patients with peripheral arterial disease to have a higher risk of various periprocedural complications. PADs, peripheral arterial disease.

Of all respondents, 78.7% (37 of 47) estimated that patients with PADs had a higher complication risk during PCI procedures or 30 days thereafter. Complications more frequently expected were: difficulties with vascular access (70.2%, 33 of 47); peripheral, noncardiac, embolic complications (61.7%, 29 of 47); bleeding at vascular access site (44.7%, 21 of 47); periprocedural myocardial infarction (MI) (27.7%, 13 of 47); bleeding unrelated to vascular access site (17.0%, 8 of 47); and vasospasm at vascular access site (17.0%, 8 of 47). Interventional cardiologists more often expected complications due to difficulties in vascular access (88.2% vs 60.0%), bleeding at vascular access site (64.7% vs 44.7%), and peripheral noncardiac embolization (82.4% vs 61.7%), when compared with general cardiologists (Fig. 2). In contrast to the respondents’ expectations, the clinical trial showed no statistically significant differences between patients with and without PADs in documented complications during the PCI procedure or the first 30 days thereafter. Bleeding at the vascular access site was present in 0.5% of patients both with and without PADs (2 of 440 and 25 of 5549, respectively). In addition, bleeding unrelated to the vascular access site occurred in 1.8% (8 of 440) of patients with PADs and in 1.4% (75 of 5549) of patients without PADs (*P* = 0.48), and a periprocedural MI occurred in 2.5% (11 of 440) and 1.4% (79 of 5549), respectively (*P* = 0.07).

### Preferences of treatment

For most respondents (80.9%, 38 of 47) the presence of PADs had an impact on the choice of vascular access, with a preference of radial arterial access in most respondents. In addition, the majority of the respondents (72.3%, 34 of 47) indicated that their choice of antiplatelet therapy was not affected by the presence of PADs.

### Clinical case

One clinical case was outlined to further investigate the cardiologists’ views on treating patients with PADs.

#### Case---impact of PADs on preferred coronary treatment

The clinical case introduced an active 70-year old man with angina pectoris, resulting from 3-vessel CAD without left main stem involvement, who had a history of invasive treatment for PADs. The presence of PADs did not have an impact on the respondents’ choice of coronary treatment in 63.8% (30 of 47), whereas 17.0% (8 of 47) of respondents were more likely to perform PCI, 12.8% (6 of 47) preferred coronary artery bypass grafting, and 6.4% (3 of 47) favored medical treatment only.

### Long-term risk of adverse events

The cardiologists’ views on the 3-year risks of complications and adverse clinical outcomes, such as bleeding, restenosis or repeated PCI, recurrent myocardial infarction, and cardiac death, were assessed in patients with and without PADs. The bleeding risk was estimated to be higher in PADs patients, which is in accordance with the results of the trials, as PCI patients with concomitant PADs had a 7.7% risk for bleeding during 3-year follow-up, compared with 5.0% risk in those without PADs (*P* = 0.014).

Similarly, the risks of MI, and restenosis or repeated PCI were estimated to be higher in PCI patients with PADs (Supplemental Table S2). The expected higher risk of MI in the PADs group is supported by the 3-year follow-up data from the clinical trials, whereas the rate of repeated revascularization showed no statistically significant difference between patients with and without PADs: in patients with PADs, 6.6% (29 of 440) experienced any MI during or after PCI, compared with 3.8% (213 of 5549) in patients without PADs (*P* = 0.005); in addition, the risk of repeated revascularization was 12.0% (53 of 440) in patients with PADs and 9.6% (532 of 5549) in patients without PADs (*P* = 0.10, Table 2).

**Table 2:** Adverse clinical events in participants in 2 all-comer PCI trials

	PADs (n = 440)	No PADs (n = 5549)	*P* value
Procedural and 30-day complications			
Bleeding at the vascular access site	2 (0.5%)	25 (0.5%)	0.94
Bleeding unrelated to the vascular access site	8 (1.8%)	75 (1.4%)	0.48
Periprocedural myocardial infarction	11 (2.5%)	79 (1.4%)	0.07
Complications until follow-up after 3 years			
Bleeding	34 (7.7%)	279 (5.0%)	0.014∗
Any myocardial infarction	29 (6.6%)	213 (3.8%)	0.005∗
Repeated revascularization	53 (12.0%)	532 (9.6%)	0.10
Cardiac mortality	18 (4.1%)	91 (1.6%)	< 0.001∗

Data expressed as number (%) unless indicated otherwise.

PADs, peripheral arterial disease; PCI, percutaneous coronary intervention.

∗ *P* < 0.05 (statistically significant).

The respondents estimated the 3-year risk of cardiac death after PCI to be between 1% and 4% in patients without PADs; in the trials that risk was 1.6% (91 of 5549). In patients with PADs, the estimated mortality risk showed a much wider range (1%-15%). The higher estimated risk of cardiac mortality in PCI patients with PADs is in line with the trial results that revealed a mortality risk of 4.1% (18 of 440) in this patient group at 3-year follow-up, which is 2.5-fold higher than in patients without PADs (*P* < 0.001).

## Discussion

### Main findings

This study is the first to evaluate cardiologists’ views on the preferred treatment and assumed post-PCI complication risk of PCI patients with concomitant PADs. In addition, the findings were compared with clinical data obtained from a pooled analysis of patient-level data from 2 large-scale clinical trials in all-comer patients who underwent PCI. The cardiologists showed substantial heterogeneity when classifying subgroups of patients as PADs patients. In addition, interventional cardiologists classified patients with an atherosclerotic stenosis of the aorta more often as PADs patients than general cardiologists. About half of cardiologists indicated an increase in concomitant PADs among PCI patients throughout the last 5 years. This increase was also observed in the clinical trials, yet the current prevalence of PADs among PCI all-comers (7.3%) was clearly lower than that estimated by cardiologists (22%). In addition, the cardiologists expected patients with PADs to have a higher risk for complications both during the PCI procedure and in the first 30 days after the procedure, but this was not observed in the clinical trial data. However, the cardiologists recognized PCI patients with PADs as having a higher risk for adverse events during 3-year follow-up, which is in line with the clinical trial data that these patients showed significantly higher rates of bleeding, MI, and cardiac mortality. Notably, for 80% of cardiologists, the presence of PADs had an impact on their choice of vascular access, but the impact of PADs on their choice of antiplatelet therapy was limited.

### Definition of PADs

The cardiologists varied widely in whom they classified as having PADs. In addition, they tended not to classify as PADs patients those who had symptoms consistent with PADs but no information on treatment, as well as patients with a history of an ischemic cerebrovascular accident but no cardiac embolic source. The heterogeneity in how cardiologists classify patients with PADs may be attributed to the use of nonuniform definitions of PADs proposed by the various medical societies. For example, the European Society of Cardiology and the European Society for Vascular Surgery define peripheral arterial disease as atherosclerotic disease that encompasses all arterial beds except for the coronary arteries and the aorta (ie, the arteries of upper and lower extremities, and the carotid, vertebral, mesenteric, and renal arteries).^16^ Yet, the American College of Cardiology Foundation and the American Heart Association consider it as an atherosclerotic disease in the arteries of the lower extremities, renal and mesenteric arteries, and the abdominal aorta,^17^ and the Canadian Cardiovascular Society and Dutch Federation of Medical Specialists focus on the arteries of the lower limbs only.^18^, ^19^, ^20^

### Prevalence of PADs among PCI patients

Over the past 5 years, about half of the cardiologists observed an increase in the prevalence of patients with concomitant PADs. This trend was also observed in the present analysis, when comparing the prevalence of PADs in participants in the 2 consecutive all-comer PCI trials BIO-RESORT and BIONYX. In The Netherlands, both the absolute number of patients with CAD and the number of patients with PADs requiring medical treatment are increasing.^3^^,^^4^^,^^21^ Therefore, it is reasonable to expect that there will be an increase in the prevalence of patients with both obstructive CAD and PADs. In our study, the cardiologists estimated that currently 22% of the PCI patients also had PADs, which is consistent with previous studies that reported a prevalence up to 19%.^1^^,^^22^, ^23^, ^24^, ^25^ The lower prevalence of PADs observed in our PCI all-comer trials may be due to differences in the definition of PADs and in the patient population examined.^6^

### Three-year adverse event risk

Our findings suggest that the responding cardiologists had a reasonable comprehension of the risk of bleeding, MI, and mortality in the PCI patients with and without PADs. Accordingly, a previous all-comers study of PCI patients showed 3-year bleeding rates of 5.0% in patients without PADs and 7.7% in those with PADs.^7^ Although most cardiologists estimated the bleeding risk after 3 years to be between 1% and 4% in PCI patients without PADs, more than half of them estimated it to be 5%-9% in patients with PADs, which is in accordance with the literature.^7^ In addition, previous research revealed a 3-year MI rate of 4.1% in PCI patients without PADs, whereas those with PADs had a higher rate of 6.4%.^6^ The cardiologists tended to estimate the MI risk in PCI patients without PADs to be somewhat lower, with about half of them estimating it to be between 1% and 2%; however, for those with PADs, half of the cardiologists correctly estimated a higher MI risk of 5%-9%. Furthermore, PCI patients with PADs have a higher 3-year cardiac mortality rate than those without PADs (4.7% vs 2.0%).^6^ Similarly, > 90% of the cardiologists estimated cardiac mortality to be 1%-4% in PCI patients without PADs and more than twice as high in those with PADs (5%-9%).^6^ A recently published study with a 10-year follow-up demonstrated a significant long-term excess mortality risk in PCI patients with concomitant PADs.^26^

Thus, patients who undergo PCI and have concomitant PADs have a higher risk for several adverse clinical events as compared with those without PADs. This was widely acknowledged by the cardiologists who responded to our survey. Given this increased risk, patients with PADs who undergo PCI should be considered a high-risk subpopulation that could benefit from tailored medical treatment. Nevertheless, our survey revealed that the cardiologists hold disparate views on the preferred treatment for patients with PADs who require PCI. This may be attributed to the somewhat limited data currently available. Therefore, this issue deserves more clinical attention and warrants further research.

### Implications of the study

Our study has shown that cardiologists classify heterogeneous groups of patients as having PADs. Unifying the definition of PADs and using the same definition in different international medical guidelines may avoid confusion and facilitate research by increasing the comparability of CAD patients with concomitant PADs evaluated in different studies. In clinical practice, a universal definition of PADs would also be useful when discussing optimal medical treatment and secondary prevention measures in heart teams and interdisciplinary discussions. In addition, our findings suggest that education about PADs and its clinical implications could be improved. Increased awareness and knowledge of the true risk of complications in CAD patients with PADs may help to tailor treatment for these patients in the future.

### Limitations

This study has some limitations. Because of the nature of our analysis, the results should be considered hypothesis-generating. The response rate to the survey (23%) was quite low, but in line with previous postal questionnaires among Dutch cardiologists, which had response rates of 10%-54%.^27^, ^28^, ^29^ The number of responding interventional cardiologists was relatively low. Yet, in The Netherlands, the majority of diagnostic coronary catheterizations are performed by general cardiologists. Nevertheless, due to the limited sample size, we cannot exclude that the respondents may be not fully representative of all cardiologists in our country. It is possible that responding cardiologists may have more interest (or expertise) in PADs than those who did not respond to the questionnaire. Furthermore, the latter may have different treatment preferences and views regarding PADs. As in other questionnaire-based studies, this bias may have affected the study findings. Despite testing the questionnaire for phrasing and order among nonparticipating cardiologists, we cannot fully exclude the possibility of some ambiguities or biases in the questions that may have influenced the respondents’ answers. We assessed not only the preferred treatment but also the respondents’ estimations and perceptions about concomitant PADs, which are subjective and impact the choice of preferred treatment. Yet, a detailed analysis of factors that may have influenced the cardiologists’ perceptions was beyond the scope of this study, but it warrants investigation in future studies. In addition, it would have been if interest to assess more in depth the reasons why several cardiologists overestimated the prevalence of PADs and the incidence of adverse events until 30 days after PCI in patients with PADs; future research should address this knowledge gap. Our findings may not be generalizable to other health-care systems with different practices and patient populations.

The number of patients with PADs in the clinical trials is underestimated, as some patients with asymptomatic or undiagnosed PADs will have been missed due to the approach by which PADs was classified (ie, based on anamnesis and medical records for confirmation). Nevertheless, the clinical relevance of asymptomatic or undiagnosed PADs, which was not assessed in this study, may be debatable. In addition, the definition of PADs may have differed between the trials and the questionnaire, as the cardiologists used their own definition of PADs, which varied between the respondents. The assessed trials enrolled the participants some years ago. Since then, there have been some improvements in treatment and risk factor modification that may have improved clinical outcome. In addition, we cannot exclude the possibility that the proportion of CAD patients with PADs has increased somewhat. We did assess discrepancies between cardiologists’ perceptions and actual clinical trial data, but we did not explore potential reasons, such as differences in local institutional standards and patient populations, or personal clinical experience.

## Conclusions

Cardiologists correctly estimated the increase of PADs over time among PCI patients, but they overestimated the current prevalence. An increased risk of adverse clinical events after PCI was correctly recognized, and concomitant PADs often affected the preferred treatment. Notably, cardiologists held disparate views on preferred coronary treatment, which may be attributed to a lack of reported data about this patient group that deserves more clinical attention and warrants future research.
