# Supplementary material for: Preferred Treatment and Expected Risk in Coronary Intervention Patients With Peripheral Arterial Disease: Cardiologists’ Views Versus Trials Data
Source: CJC Open. 2025 Mar 31;7(6):799–806. doi: 10.1016/j.cjco.2025.03.018 (PMC12198597; doi:10.1016/j.cjco.2025.03.018)
Supplement: Supplementary Material [file mmc1.pdf]

Preferred Treatment and Expected Risk in Coronary Intervention Patients with Peripheral Arterial Disease: Cardiologists' Views Versus Trials Data

Tineke H. Pinxterhuis, MD[a,b], Clemens von Birgelen, MD PhD[a,b*], Eline H. Ploumen, MD PhD[a,b], Daphne van Vliet, MD[a,b], Marlies M. Kok, MD PhD[a], Rosaly A. Buiten, MD PhD[a], Liefke C. van der Heijden, MD PhD[a], Paolo Zocca, MD PhD[a], Carine J.M. Doggen, PhD[b,c]

a. Department of Cardiology, Thoraxcentrum Twente, Medisch Spectrum Twente, Enschede, the Netherlands
b. Health Technology and Services Research, Faculty BMS, Technical Medical Centre, University of Twente, Enschede, the Netherlands
c. Clinical Research Center, Rijnstate Hospital, Arnhem, the Netherlands

SUPPLEMENTARY MATERIAL

Index

Supplemental Appendix S1:Questionnaire ________________________ *page 2*

*Supplemental Table S1: Responses of cardiologists on definition of peripheral arterial disease, treatment preferences and procedural complications*________________________ *page 8*

Supplemental Table S2: Complication risks of patients with and without PADs as estimated by cardiologists ________________________ *page 11*

Supplemental Appendix S1: Questionnaire

General questions

1. In which type hospital are you working? (multiple answers possible)
   - Non-academic hospital without PCI and without cardiac surgery
   - Non-academic hospital with PCI but without cardiac surgery
   - Non-academic hospital with PCI and with cardiac surgery
   - Academic hospital
   - Other, ………………………………………………………..
2. How do you consider yourself?
   - Woman
   - Man
   - Other
   - I'd rather not say
3. What is your age?
   - <29 years
   - 30-39 years
   - 40-49 years
   - 50-59 years
   - >60 years
4. Are you an interventional cardiologist?
   - Yes
   - No
5. Do you perform PCIs?
   - Yes
   - No
6. How many years do you perform PCI?
   ………………………………………………………..
7. How many PCIs per year do you perform on average?
   ………………………………………………………..
8. Which percentage of patients who undergo treatment with PCI have peripheral arterial disease as co-morbidity?
   ………………………………………………………..
9. Has the number of PCI patients with peripheral vascular disease increased in your hospital in the past 5 years?
   - Yes
   - No

Peripheral arterial disease

10. Which patients would you classify as 'patients having peripheral arterial disease'? (multiple answers possible)

    Patients with:
    - Intermittent claudication treated by a vascular surgeon
    - Intermittent claudication treated by the general practitioner
    - Symptoms consistent with intermittent claudication without information of the vascular surgeon or general practitioner
    - A known stenosis in the mesenteric artery
    - An ischemic cerebrovascular accident without cardiac source of embolism
    - Known stenosis in the vertebral artery or carotid artery
    - Known stenosis in the arteries of the upper extremities
    - Patients with an atherosclerotic stenosis in the aorta with symptoms or which require treatment

Case 1:

Imagine a 70-year-old patient being discussed during Heart Team discussion for 3-vessel disease. He walks regularly and has presented with stable angina pectoris. CAG shows 3-vessel disease without involvement of the main stem (anatomically, treatment by both PCI and CABG is possible). He has a history of invasive treatment for peripheral vascular disease.

11. Does the fact that a patient has a history of peripheral arterial disease, affects your treatment?
    - Yes
    - No

    How has a history of peripheral arterial disease impact on your treatment? I would rather choose:
    - CABG
    - PCI
    - Treatment with drugs

Case 2:* **On the advice of the reviewers, the case was removed from the manuscript.**

Imagine a 70-year-old patient, who is known to the vascular surgeon with peripheral arterial disease and who underwent a PCI due to non-STEMI 1 month ago. He is prescribed DAPT (ticagrelor or prasugrel + Ascal) for 1 year. He comes to the ER with a gastro-intestinal bleed and you are called by the gastroenterologist for advice.

12. Which advice do you give?
    - Leave policy unchanged: maintain ticagrelor/prasugrel + Ascal and continue DAPT for 1 year

- Shorter DAPT duration: maintain ticagrelor/prasugrel + Ascal and continue DAPT for 6 months
- Less potent DAPT: replace ticagrelor/prasugrel with clopidogrel and continue DAPT for 1 year
- Less potent DAPT and shorter DAPT duration: replace ticagrelor/prasugrel with clopidogrel and continue DAPT for 6 months
- Discontinue DAPT after 4 weeks: replace ticagrelor/prasugrel + Ascal with P2Y12 inhibitor monotherapy
- Other, namely ………………………………………………………..

13. Do you have the impression that PCI patients with peripheral arterial disease have an increased risk for complications during PCI or 30 days afterwards?
    - Yes
    - No

    What complications do patients with peripheral arterial disease have an increased risk of? (multiple answers possible)
    - Difficult arterial access site
    - Vascular spasm of the arterial access site
    - Bleeding from the arterial access site
    - Bleeding, not related to the arterial access site
    - Peri-procedural myocardial infarction
    - Peripheral non-cardiac embolic complications (e.g. ischemic stroke, peripheral vascular occlusion)
    - Other, namely ………………………………………………………..

Imagine a PCI has to be performed in a patients with peripheral arterial disease.

14. Has this information impact on your preference for vascular access site?
    - Yes
    - No
15. Which access site would you prefer in patients with peripheral arterial disease?
    - A. radialis
    - A. ulnaris
    - A. brachialis
    - A. femoralis
16. Has peripheral arterial disease impact on your choice of antiplatelet therapy?
    - Yes
    - No

17. How would you change antiplatelet therapy in patients with peripheral arterial disease with regard to patients without peripheral arterial disease?
    - o Less potent DAPT: clopidogrel instead of ticagrelor/prasugrel
    - o More potent DAPT: ticagrelor/prasugrel instead of clopidogrel
    - o Longer treatment with DAPT: STEMI patients longer than 1 year DAPT treatment
    - o Shorter treatment of DAPT: STEMI patients shorter than 1 year DAPT treatment
    - o Others, ……………………………………………………………..

18. What is the risk for **bleeding** in patients <u>without</u> peripheral arterial disease in the first 3-years after PCI?
    - o 1-2%
    - o 3-4%
    - o 5-9%
    - o 10-14%
    - o ≥15%
19. Do you think that patients with peripheral arterial disease have a higher risk for **bleeding** in the first 3-years after PCI
    - o Yes
    - o No
20. What is the risk for **bleeding** in patients <u>with</u> peripheral arterial disease in the first 3-years after PCI?
    - o 1-2%
    - o 3-4%
    - o 5-9%
    - o 10-14%
    - o ≥15%
21. What is the risk for **restenosis and re-PCI of the target vessel** in patients <u>without</u> peripheral arterial disease in the first 3-years after PCI?
    - o 1-2%
    - o 3-4%
    - o 5-9%
    - o 10-14%
    - o ≥15%
22. Do you think that patients with peripheral arterial disease have a higher risk for **restenosis and re-PCI of the target vessel** in the first 3-years after PCI.
    - o Yes

- No

23. What is the risk for **restenosis and re-PCI of the target vessel** in patients with peripheral arterial disease in the first 3-years after PCI?
    - 1-2%
    - 3-4%
    - 5-9%
    - 10-14%
    - ≥15%
24. What is the risk for **myocardial infarction** in patients without peripheral arterial disease in the first 3-years after PCI?
    - 1-2%
    - 3-4%
    - 5-9%
    - 10-14%
    - ≥15%
25. Do you think that patients with peripheral arterial disease have a higher risk for **myocardial infarction** in the first 3-years after PCI
    - Yes
    - No
26. What is the risk for **myocardial infarction** in patients with peripheral arterial disease in the first 3-years after PCI?
    - 1-2%
    - 3-4%
    - 5-9%
    - 10-14%
    - ≥15%
27. What is the risk for **cardiac death** in patients without peripheral arterial disease in the first 3-years after PCI?
    - 1-2%
    - 3-4%
    - 5-9%
    - 10-14%
    - ≥15%
28. Do you think that patients with peripheral arterial disease have a higher risk for **cardiac death** in the first 3-years after PCI
    - Yes
    - No

29. What is the risk for **cardiac death** in patients with peripheral arterial disease in the first 3-years after PCI?
    - 1-2%
    - 3-4%
    - 5-9%
    - 10-14%
    - ≥15%

Finally: Are there any topics you would have liked to discuss in the context of peripheral artery disease and PCI that have not been covered?

…………………………………………………………..

Supplemental Table S1: Responses of cardiologists on definition of peripheral arterial disease, treatment preferences, and procedural complications

Patients having peripheral arterial disease	
Intermittent claudication treated by a vascular surgeon	45 (95.7)
Intermittent claudication treated by the general practitioner	37 (78.7)
Symptoms consistent with intermittent claudication without information of the vascular surgeon or general practitioner	12 (25.5)
A known stenosis in the mesenteric artery	41 (87.2)
An ischemic cerebrovascular accident without cardiac source of embolism	26 (55.3)
Known stenosis in the vertebral artery or carotid artery	36 (76.6)
Known stenosis in the arteries of the upper extremities	41 (87.2)
Patients with an atherosclerotic stenosis in the aorta with symptoms or which require treatment	40 (85.1)
Case 1: 70-year-old patient with peripheral arterial disease, discussed during Heart Team discussion for 3-vessel disease.	
The fact that a patient has a history of peripheral arterial disease, affects your treatment	17 (36.2)
I would rather choose:	
CABG	6 (12.8)
PCI	8 (17.0)
Treatment with drugs	3 (6.4)
Case 2: 70-year-old patient with peripheral arterial disease who underwent PCI due to non-STEMI 1 month ago. Now presenting with a gastro-intestinal bleeding. Which advice do you give?	
Leave policy unchanged: maintain ticagrelor/prasugrel + Ascal and continue DAPT for 1 year	1 (2.1)

Shorter DAPT duration: maintain ticagrelor/prasugrel + Ascal and continue DAPT for 6 months	3 (6.4)
Less potent DAPT: replace ticagrelor/prasugrel with clopidogrel and continue DAPT for 1 year	7 (14.9)
Less potent DAPT and shorter DAPT duration: replace ticagrelor/prasugrel with clopidogrel and continue DAPT for 6 months	17 (36.2)
Discontinue DAPT after 4 weeks: replace ticagrelor/prasugrel + Ascal with P2Y12 inhibitor monotherapy	9 (19.1)
Other	10 (21.3)
PCI patients with peripheral arterial disease have an increased risk for complications during PCI or 30 days afterwards	37 (78.7)
Difficult arterial access site	33 (70.2)
Vascular spasm of the arterial access site	8 (17.0)
Bleeding from the arterial access site	21 (44.7)
Bleeding, not related to the arterial access site	8 (17.0)
Peri-procedural myocardial infarction	13 (27.7)
Peripheral non-cardiac embolic complications (e.g. ischemic stroke, peripheral vascular occlusion)	29 (61.7)
The presence of peripheral arterial disease has impact on the preference for vascular access site	38 (80.9)
Which access site would you prefer in patients with peripheral arterial disease?	
A. radialis	38 (80.9)
A. ulnaris	0
A. brachialis	0
A. femoralis	0
The presence of peripheral arterial disease has impact on the choice of antiplatelet therapy	13 (27.7)

Data are n (%), unless otherwise indicated.

Abbreviations: CABG= Coronary Artery Bypass Grafting, DAPT= Dual Antiplatelet Therapy, PADs=peripheral arterial disease; PCI=percutaneous coronary intervention, STEMI; ST-segmented elevated myocardial infarction

Supplemental Table S2: Complication risks of patients with and without PADs as estimated by cardiologists

	**1-2%**	**3-4%**	**5-9%**	**10-14%**	**>15%**
Patients without PADs					
Bleeding	15 (31.9)	21 (44.7)	9 (19.1)	1 (2.1)	0
Repeated revascularization	15 (31.9)	23 (48.9)	7 (14.9)	1 (2.1)	0
Myocardial infarction	21 (44.7)	20 (42.6)	3 (6.4)	1 (2.1)	1 (2.1)
Cardiac mortality	27 (57.4)	16 (34.0)	1 (2.1)	1 (2.1)	1 (2.1)
Patients with PADs					
Bleeding	4 (8.5)	7 (14.9)	22 (46.8)	9 (19.1)	4 (8.5)
Repeated revascularization	1 (2.1)	7 (14.9)	20 (42.6)	14 (29.8)	4 (8.5)
Myocardial infarction	1 (2.1)	10 (21.2)	18 (38.3)	14 (29.8)	3 (6.4)
Cardiac mortality	10 (21.2)	13 (27.7)	15 (31.9)	5 (10.6)	2 (4.3)

Data are n (%), unless otherwise indicated.
Abbreviations: PADs = peripheral arterial disease
